# Cytogenetic and Molecular Analysis of a “Double‐Hit” *RUNX1* Including a *RUNX1* p.Trp279* and a Cryptic Novel t(6;21)(q25;q22)/*RUNX1*::*ARID1B* in Acute Myeloid Leukemia

**DOI:** 10.1002/gcc.70148

**Published:** 2026-06-12

**Authors:** Rolando García, Jing Xu, Lan Yu, Kalayarasan Srinivasan, Sharon Koorse Germans, Olga Weinberg, Franklin Fuda, Weina Chen, Prasad Koduru

**Affiliations:** ^1^ Department of Pathology UT Southwestern Medical Center Dallas Texas USA

## Abstract

**Introduction:**

Alterations involving *RUNX1* are recurrent in hematologic malignancies and contribute to disease pathogenesis via dysregulation of transcriptional factors essential for hematopoiesis. Here, we report an acquired alteration in both alleles of *RUNX1*; one is a truncating mutation and the second is a novel *RUNX1::ARID1B* identified in acute myeloid leukemia.

**Methods:**

Bone marrow samples were assessed by morphologic examination and flow cytometry. Cytogenetic analysis was performed using conventional G‐banded karyotyping and fluorescence in situ hybridization (FISH). Molecular profiling was performed using next‐generation sequencing (NGS) for single nucleotide changes, copy number variations, gene fusions, and expression.

**Results:**

Morphology showed large‐sized myeloblasts that are CD34+ and CD45+ (dim) consistent with acute myeloid leukemia. Genetic analysis of diagnostic specimen showed normal karyotype and FISH results but detected mutations in *IDH1* and *RUNX1*. Cytogenetic analysis of a specimen at relapse showed complex abnormal karyotype and FISH detected *RUNX1* rearrangement with 6q25. NGS identified this rearrangement as *RUNX1::ARID1B*. Thus, at this stage leukemia cells had “double‐hit” abnormality in *RUNX1*. Gene transcript evaluation showed elevated levels of transcripts of both *RUNX1* and *ARIDB1*.

**Conclusion:**

This study expands the spectrum of *RUNX1* fusions and highlights the integral diagnostic value of morphology, flow cytometry, cytogenetics, FISH, and NGS analyses for broad structural variant detection in clinical practice. Furthermore, the truncating mutation in one allele of *RUNX1* and *RUNX1*::*ARID1B* of the second allele detected with advanced disease suggests the possibility of combined transcriptional and chromatin regulatory alterations in disease recurrence in the patient.

## 1 Introduction

*RUNX1* (at 21q22) encodes the alpha subunit of the core binding factor (CBF) transcription factor which binds to promoters and enhancers. The RUNX1 protein plays an important role in hematopoiesis, contributing to stem cell generation and differentiation into myeloid and lymphoid precursors [1]. The role of the RUNX1 protein depends on the quantity and characteristics of the different proteins that interact with it. RUNX1 protein encompasses several key functional domains: an evolutionary conserved 128‐amino acid runt homology domain (RHD), a 168‐sequence transcription activation domain (TAD), and a conserved five amino acid sequence of VWRPY at the C‐terminal end [2, 3, 4, 5, 6, 7]. Three main *RUNX1* transcript isoforms (*RUNX1a*, *b* and *c*) are generated from proximal and distal promoters that are 160 kb apart, but all three contain the RHD domain. Due to its significant involvement in hematopoietic differentiation, several chromosome translocations involving *RUNX1* have been detected in hematological neoplasms. At present, approximately 70 different translocations have been identified and about 67% of these involved a partner gene [8]. The structure and function of *RUNX1* fusions may be categorized into five groups: (1) in‐frame fusion maintaining the RHD domain but lacking the TAD domain [t(8;21)(q22;a22)/*RUNX1::RUNX1T1*], (2) truncated fusion gene with part of the RHD but losing the TAD domain [t(1;21)(q21;q22)/*RUNX1*::*ZNF687*], (3) fusion gene with RHD but not the TAD domain [t(3;21)(q26;q22)/*RUNX1::RPL22L1*], (4) rearrangement not retaining the RHD [inv(21)(q21q22)/*USP16*::*RUNX1*], and (5) in‐frame gene rearrangement retaining both RHD and TAD domains [t(12;21)(p13;q22)/*ETV6*::*RUNX1*] [5]. The fusion product might play a role in the development of leukemia by impacting multiple signaling pathways, such as *RAS*, receptor tyrosine kinase (RTK), and GATA. Additionally, it may collaborate with other genetic sequence variants or chromosome abnormalities [9, 10, 11, 12].

In addition to fusion genes, somatic sequence variants in *RUNX1* are distributed throughout the gene; however, most pathogenic or likely pathogenic variants are mapped within the RHD domain [13]. Allelic *RUNX1* abnormalities alone do not lead to disease development; additional somatic genetic alterations are required. For instance, mutations in the second *RUNX1* allele have been observed during the transformation of familial platelet disorder (FPD) to AML [14].

Switch/sugar non‐fermenting (SWI/SNF) chromatin remodeling complex is involved in normal hematopoiesis. *ARID1B* encodes a subunit of SWI/SNF and has a crucial role in myelopoiesis. Alterations in *ARID1B* can disrupt SWI/SNF function, resulting in epigenetic dysregulation and development of cancer [15].

Here we present a case of AML featuring pathogenic variants in the *RUNX1* and *IDH1* at diagnosis and subsequently acquired a novel t(6;21)(q25;q22)/*RUNX1*::*ARID1B*, resulting in a genomic “double hit” to *RUNX1* and disease relapse.

## 2 Materials and Methods

This study was reviewed and approved by the Institutional Review Board of UT Southwestern.

### 2.1 Morphology and Immunohistochemistry

Bone marrow (BM) aspirate smear was stained with Wright‐Giemsa, and the routine H&E‐stained BM biopsies were prepared from formalin‐fixed, paraffin‐embedded (FFPE) blocks for histological evaluation. Immunohistochemical analysis was performed on tissue block sections using standard procedures and a panel of monoclonal antibodies for CD20, CD3, CD138, CD34, CD117, CD42b, CD71, MPO following standard protocols [16].

### 2.2 Flow Cytometry

Flow cytometry of the diagnostic BM sample was completed by Neo Genomics Laboratory (Fort Meyers, FL) following their protocol. Flow cytometric study of the relapsed BM at our institution was performed on a 10‐color BD FACSCanto flow cytometer (BD Biosciences, San Jose, CA) and analyzed using cluster analysis with Cytopaint Classic software (Leukobyte), as described previously [17]. A panel of comprehensive lymphoid, myelomonocytic, and immature cell markers was used, including CD2, CD3, CD4, CD5, CD7, CD8, CD10, CD11b, CD13, CD14, CD15, CD16, CD19, CD20, CD22, CD33, CD34, CD36, CD38, CD45, CD56, CD64, CD117, CD123, HLA‐DR, mKappa, mLambda.

### 2.3 Chromosome Analysis and FISH

Cytogenetic analysis and FISH (AML panel—*RUNX1‐RUNX1T1*, *KMT2A* break‐apart, *PML‐RARA* and the *CBFB* break‐apart, MDS panel—hTERT, RPS14 LSI‐*EGR1*, CEP7, D7S2929, D7S2460, Cep8, D20S108) of diagnostic BM was performed at Neo Genomics Laboratories following their protocols. Relapsed BM was cultured for 24 and 48 h, exposed to colcemid for 20 min, hypotonic solution for 30 min, and harvested following conventional protocol for G‐banded chromosome analysis. G‐banded metaphases were analyzed and described as per ISCN 2024 [18]. FISH was then performed on both interphase nuclei and abnormal metaphase spreads to confirm gene rearrangement. FISH probes used on the relapsed sample included: *RUNX1‐RUNX1T1*, *BCR‐ABL1*, *KMT2A* break‐apart, *PML‐RARA* and the *CBFB* break‐apart (Abott Molecular, Abott Park, IL).

### 2.4 NGS

NGS on the diagnostic BM specimen was performed at Neo‐Genomics Laboratories as per their protocol. For NGS of the relapsed BM, DNA and RNA were isolated from BM clot sections with paired germline specimen from saliva. Sequencing libraries were generated using Kapa Biosystems and Illumina chemistry. A custom panel of probes was used to produce an enriched DNA library containing all exons from 183 genes that are known to be mutated in myeloid or lymphoid tumors, along with RNA library from over 1500 genes for fusion detection. Variants generated were annotated using ANSWER annotation software for electronic reporting [19]. Gene transcription profile of selected genes was also generated using ANSWER. Full details of the genes tested are available at https://ngsclialab.pages.biohpc.swmed.edu/gene‐panel‐search‐html/.

### 2.5 RNA Extraction, RT‐PCR, and Sanger Sequencing

RNA was extracted from BM aspirate using the Maxwell RSC simplyRNA Blood Kit (Promega Corp., Madison, WI). Five hundred nanograms of RNA was subjected to reverse transcript using iScript cDNA Synthesis Kit (Bio‐Rad Laboratories, Fort Worth, TX). The synthesized first‐strand cDNA was amplified with designed primers (Supplemental Table S1). PCR amplification was performed for 45 cycles at 95°C for 15 s, 63°C for 15 s, and 72°C for 30 s. After amplification, the PCR product was purified using ExoSAP‐IT Express reagent (Thermo Fisher Scientific, Waltham, MA) and was sequenced by the dideoxy chain termination method using BigDye Terminator v3.1 Cycle Sequencing Kit and ABI 3500XL Genetic Analyzer (Thermo Fisher Scientific, Waltham, MA).

To further explore the impact of the *RUNX1* truncation and *RUNX1*::*ARIDB1*, RUNX1 and ARIDB1 transcription were evaluated using box and whisker plots. The analysis was based on FPKM (fragments per kilobase millions—log2) and compared against clinical cases of the same tissue type [20].

## 3 Results

### 3.1 Morphology, Immunohistochemistry, and Flow Cytometry

At presentation (June 2021) the patient, a 77‐year‐old female, had 1.58 k/ul WBC, 11.6 g/dL hemoglobin, 93.8 fl MCV and 155 k/ul platelets. BM aspirate smear was hyper‐cellular (50%) and showed 3% myelocytes, 38% erythrocytes, 3% monocytes, 5% lymphocytes and 51% blasts. Blasts had cytoplasmic vacuoles, irregular nuclei and scant cytoplasm (Figure S1). Core biopsy sections and clot sections had 50% blasts that were CD34+, CD117+; a diagnosis of acute myeloid leukemia (AML) was made. At relapse (January 2024) morphologically BM blasts appeared similarly to the blasts in the diagnostic specimen.

Flow cytometry of BM aspirate at diagnosis revealed that the blasts were CD34+ mod, CD45+‐dim, CD13+, CD38+, CD117+, HLD‐DR+, cMPO+ (small subset). At relapse, blasts were CD7 (small subset dim+), CD11b (−), CD13 (partial dim+), CD14 (−), CD15 (partial+), CD16 (−), CD33 (partial dim+), CD34(+), CD36(−), CD38(variably+), CD45(−), CD56(partial+), CD64(predominantly‐), CD117(+), CD123(−), and HLA‐DR+ (predominantly‐). Flow cytometry plots at initial presentation and at clonal progression are in Figure S2.

### 3.2 Cytogenetics, FISH, and Molecular Studies

Genetic studies on a diagnostic specimen (performed at Neo Genomics laboratories) revealed a normal female karyotype with normal FISH for AML and MDS probes. NGS detected one pathogenic sequence variant in *IDH1* (c.394C>G p.Arg132Gly, VAF 39%) and one likely pathogenic sequence variant in *RUNX1* (c.837G>A, p.Trp279*, VAF 30%); no gene fusions were identified (Figure S3).

**FIGURE 1:**
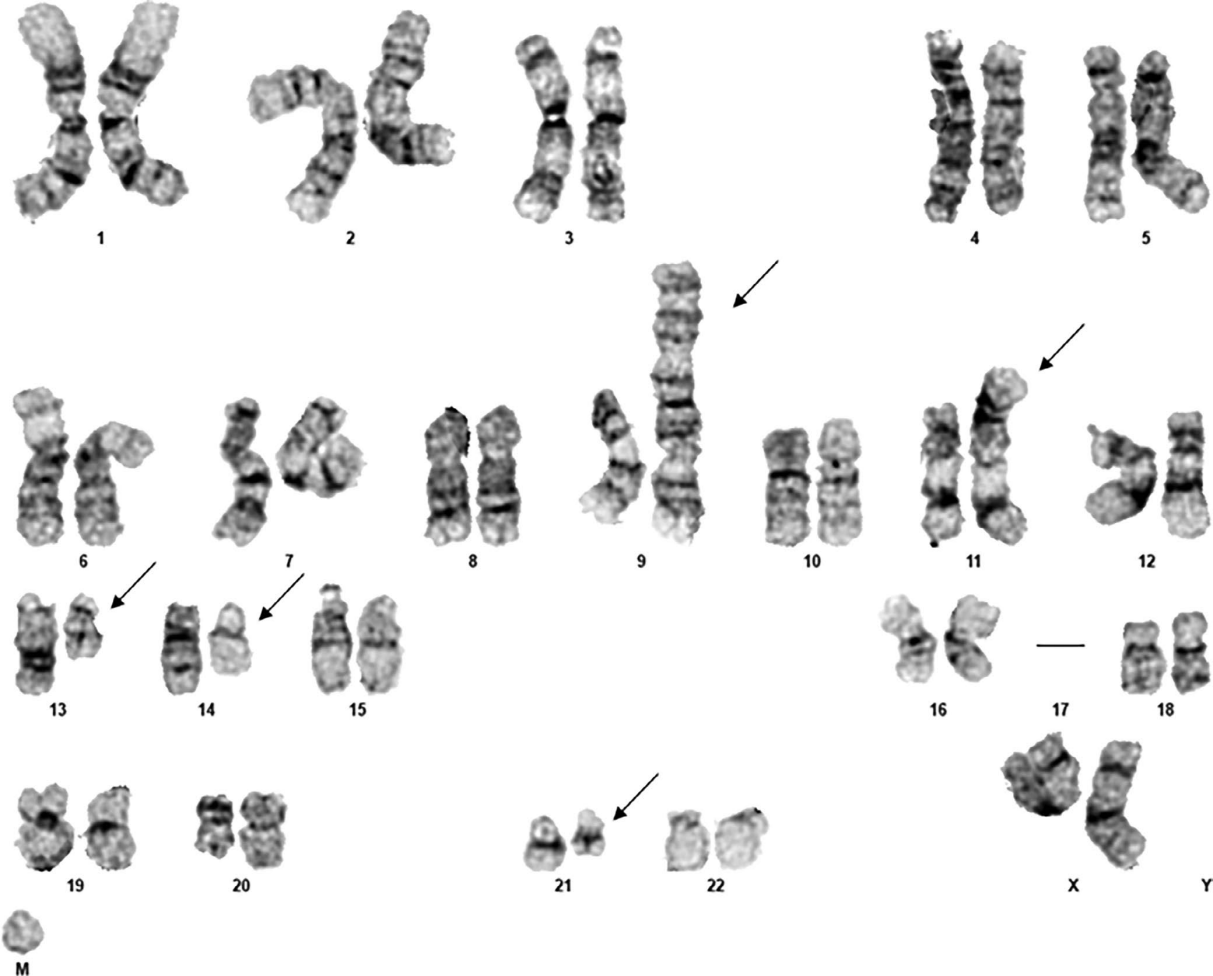
G‐banded karyogram in AML tumor cells in bone marrow sample. Abnormalities are illustrated at black arrows and marker chromosome depicted below karyogram (see text for full description of abnormalities identified by G‐banded chromosomes).

**FIGURE 2:**
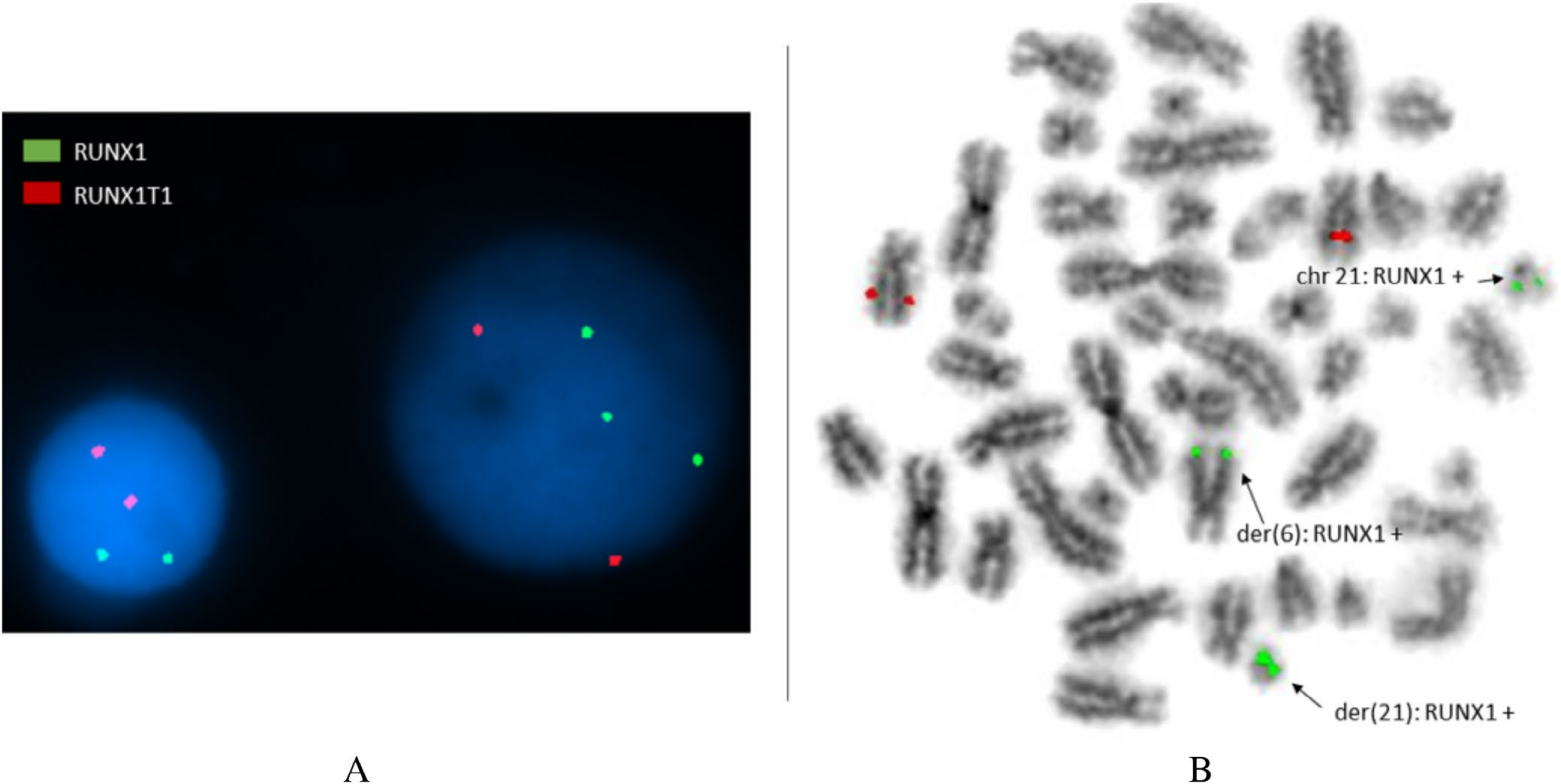
FISH performed on tumor sample. The interphase FISH image in the panel (A) depicts a normal cell with two red and two green signals, alongside an abnormal cell exhibiting an additional signal for the *RUNX1* gene, indicating a potential translocation event. (B) Subsequent hybridization of an abnormal metaphase cell with the *RUNX1/RUNX1T1* probe set revealed the extra *RUNX1* signal hybridized to the terminal end of the long arm of chromosome 6.

BM plasma had markedly elevated D‐2‐hydroxyglutarate consistent with the presence of *IDH1*+ cells. BM at relapse had complex abnormalities—45,XX, der(9;17)(17qter‐ > 17p13::17q11.2‐ > 17q25::?::9p24‐ > 9qter), t(11;13)(p13;q12), add(14)(q11.2), −17,del(21) (q22q22), +mar [20] (Figure 1). Interphase FISH detected three copies for *RUNX1* [176/200 nuclei, Figure 2A,B]. Hybridization with *RUNX1* probe on abnormal metaphases detected one signal at 21q22 and a rearranged signal at 6q25 (Figure 2B); therefore, the significant findings in this karyotype are a cryptic t(6;21)(q25;q22) and a rearranged *RUNX1*.

**FIGURE 3:**
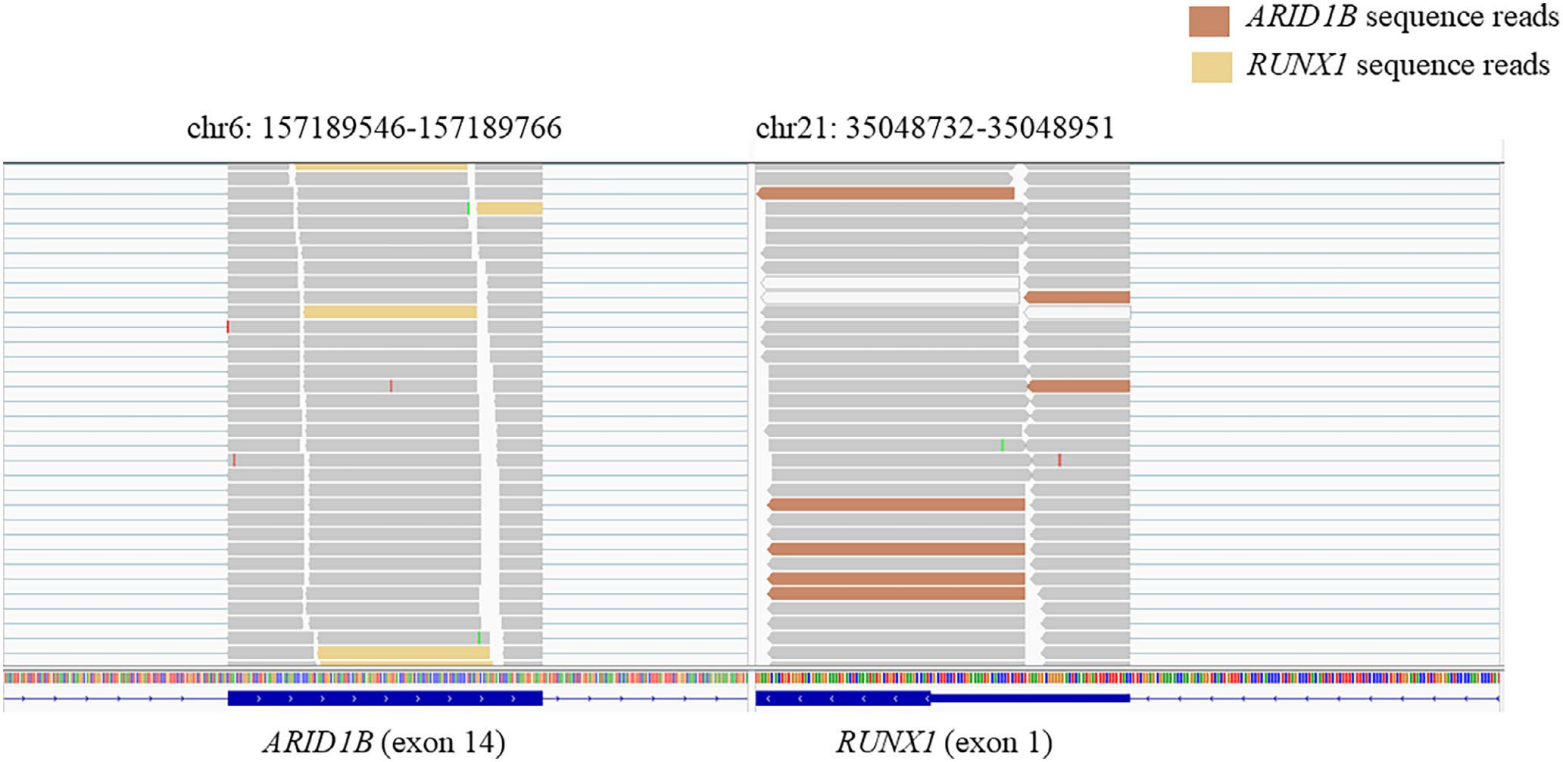
Visualization of the *RUNX1*::*ARID1B* fusion detected by NGS, as depicted in the Integrative Genomics Viewer (IGV). Split reads spanning *RUNX1* and *ARID1B* breakpoints, with reads transitioning from *ARID1B* (left) and *RUNX1* (right). The breakpoint confirms fusion of *ARID1B* exon 14 to the 5′ of *RUNX1* exon 1.

**FIGURE 4:**
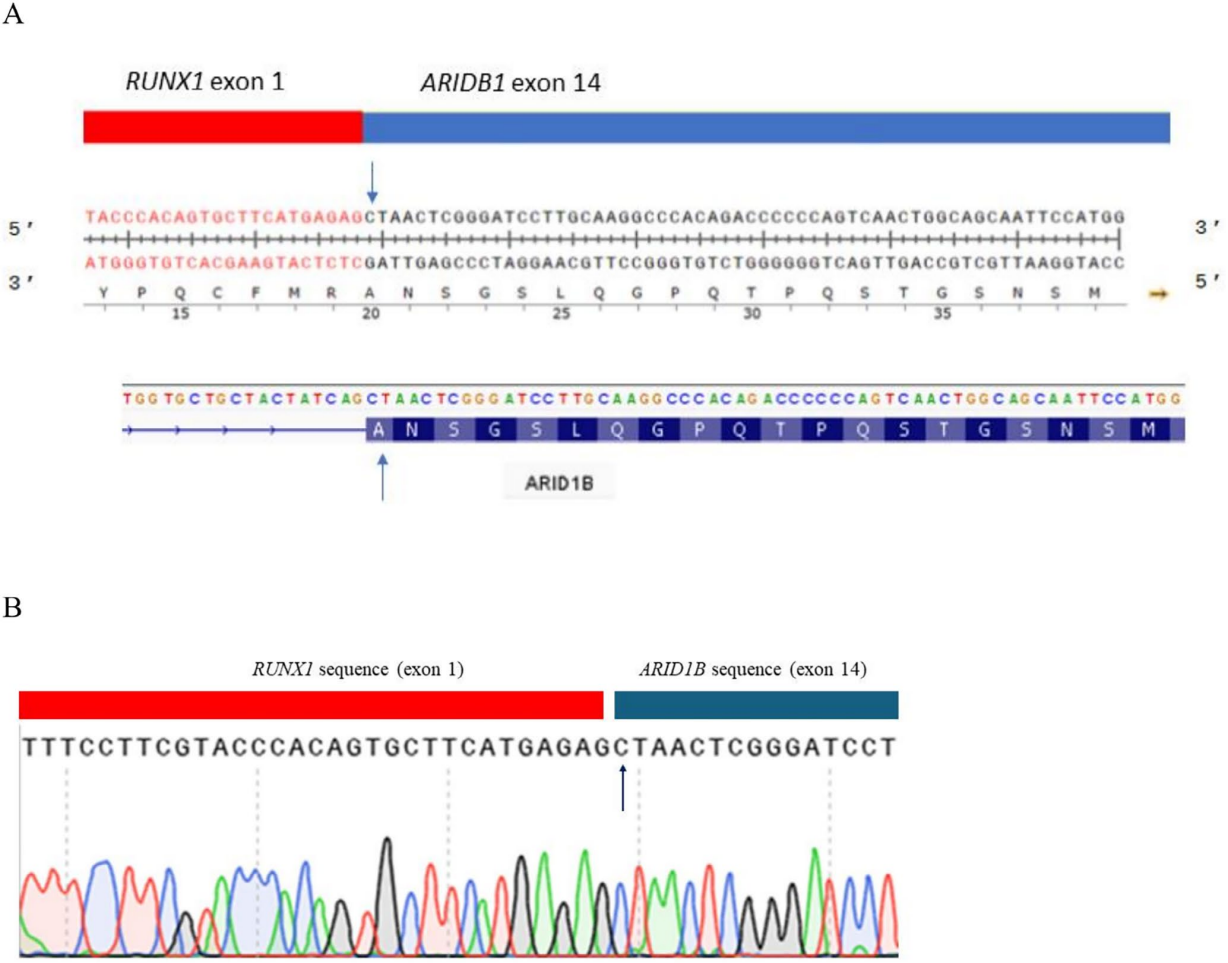
Confirmation of the fusion sequence via Sanger sequencing, illustrated by the perfect alignment of sequencing data with the fusion sequence identified through NGS analysis. (A) Fusion sequence as identified by NGS and panel (B) shows the Sanger DNA sequencing data. Colored waves represent the sanger base sequence.

The relapsed BM by NGS showed the original *IDH1* (VAF 38%) and *RUNX1* (VAF 37%) mutations, and additional molecular abnormalities in *ASXL1* (c.1934dupG p.Gly646fs, pathogenic, VAF 33%), *DNMT3A* (c.2597 + 1G > A, pathogenic, VAF 37%), and *BAX* (c.509G > A p.Trp170*, likely pathogenic, VAF 41%). RNA fusion NGS detected an in‐frame *RUNX1*::*ARIDB1* (Figure 3), consistent with the cryptic t(6;21). Sanger sequencing of RT‐PCR product mapped fusion of exon 1 of *RUNX1* with exon 14 of *ARID1B* (Figure 4). Therefore, the relapsed leukemia carried “double‐hit” lesions in *RUNX1*: the initially detected *RUNX1* c.837G > A p.Trp279* and a *RUNX1*::*ARID1B*. Other copy number variants (CNVs) that were detected in the relapsed sample were hemizygous deletions in *JAK2*: chr 9p24.1 (5071960‐5 082 395), *WTI*: chr11p15.5‐p13 (150500‐35 970 522), *NF1*: chr17q11.2 (30792839‐31 223 625), and *U2AF1*: chr21q22.3 (41787026‐44 924 571); these correlated with the karyotypic structural abnormalities affecting 9p, 11p, 17 and 21q detected in this sample.

**FIGURE 5:**
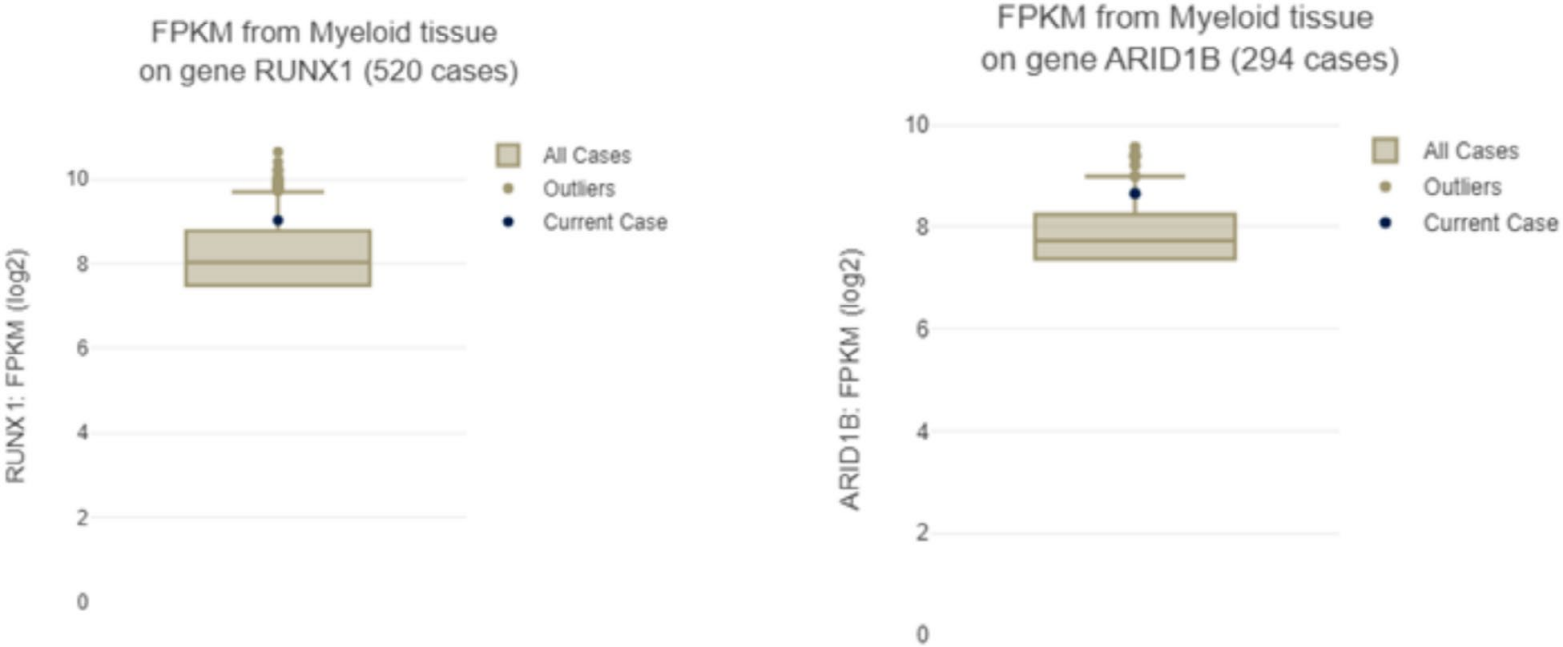
Box and whisker plots illustrating RNA expression of tumor sample from (1/2/2024) using FPKM log2 and compared with 520 myeloid tissue cases for *RUNX1* (left)and 294 cases for *ARIDB1* (right).

RNA analysis of relapsed BM showed higher transcription for *RUNX1* compared to median of 520 myeloid cases (9.02 vs. 8.03). Similarly, ARIDB1 transcripts were also higher compared to the median of 294 myeloid cases (8.65 vs. 7.72) (Figure 5). Hence, the increased abundance of RUNX1 and ARIDB1 transcripts could result directly from the fusion event. Alternatively, the truncated RUNX1 gene on the second allele might exert a dominant negative effect, triggering compensatory mechanisms. The reciprocal fusion is on positive strand and is predicted to be inactive.

### 3.3 Management

Patient underwent treatment with Venetoclax and Azacitidine (VEN‐AZA) therapy (VEN from 20 mg Day 1–7, 50 mg Day 8–14, 100 mg Day 15–21, and 200 mg Day 22–28) on 7/6/21; cycle 2 was on 8/2/21. Patient received two cycles without events. She was diagnosed with COVID on 9/4/21 after being presented with febrile neutropenia. She was treated with Regeneron. A repeat BM evaluation 3 months into therapy showed normocellular trilineage hematopoiesis; flow cytometry was negative, cytogenetics and FISH results stayed normal; however, the *IDH1* pathogenic sequence variant was detected by PCR. She continued with VEN‐AZA. BM after six cycles showed complete remission; the *IDH1* sequence variant was undetectable at this time and BM plasma showed normal range d‐2‐hydroxyglutarate (97 ng/mL, normal 18–263 ng/mL). Patient was lost to follow up from 2/28/2022 to 4/27/2023. Back in the clinic, morphology detected < 5% blasts; NGS analysis detected a *DNMT3A* mutation (c.2597 + 1G>A, 29.5% VAF), and the original *IDH1* and *RUNX1* mutations. BM plasma had markedly elevated d‐2‐hydroxyglutarate (2915 ng/mL), consistent with leukemia relapse. Patient lost for follow up again until 1/02/2024. Back in the clinic, BM showed 43% blasts and 38.7% circulating blasts with complex karyotypic abnormalities described above. Patient was started on hydrea and subsequently on Azacitidine + Ivosedinib on 1/17/2024. After receiving treatment, the patient passed away 7 months later.

## 4 Discussion

Herein we present an AML with *IDH1* [p.Arg132Gly] and *RUNX1* [p.Trp279*] sequence variants at diagnosis that later progressed to a “double‐hit” *RUNX1* alteration by acquiring a new cryptic t(6;21)(q25;q22)[*RUNX1* exon 1::ARIDB1 exon 14]. As detected in the diagnostic specimen of this patient, *IDH1* (and *IDH2*) mutations are most common in patients with cytogenetically normal AML [21]. The p.Arg132Gly variant in *IDH1* lies within the enzyme's active site and results in a gain‐of‐function and an abnormal production of 2‐hydroxyglutarate (2HG) [22]. This pathogenic effect is consistent with the elevation of 2HG observed in BM plasma.

*RUNX1* encodes different isoforms *RUNX1a, RUNX1b*, and *RUNX1c* and these are generated from two different promoters p1 and p2, all sharing the conserved RHD which is necessary for DNA and CBFβ binding [23]. *RUNX1a* binds DNA with stronger affinity and acts as a suppressor to the *RUNX1b* transcriptional activation [24]. *RUNX1* is often mutated in AML [25], and a substantial number of them are truncating mutations that worsen the transcriptional activity of the protein. The *RUNX1* p.Trp279* variant detected in diagnostic leukemia cells is embedded within the TAD domain. This mutation leads to a loss‐of‐function of RUNX1 and plays a pathogenic role in leukemogenesis [26]. Previous studies have provided evidence that truncated RUNX1 protein produced by chromosomal translocations can act in a dominant negative manner and further enhance leukemogenesis [27, 28]. *ARID1B* is a subunit of the SWI/SNF nucleosome complex, a highly conserved regulator 3, lines 2–7, and para in chromatin remodeling to control gene expression. Alterations in *ARID1B* can disrupt SWI/SNF function, resulting in epigenetic dysregulation and development of cancer, including AML [15]. Over 70 fusion partners of *RUNX1* have been reported in hematologic cancers [29], however, the *RUNX1::ARID1B* has not been described previously in AML. Sanger sequencing of cDNA of the relapsed BM confirmed fusion between *RUNX1‐*exon 1 and *ARID1B*‐exon 14, it is predicted to be an in‐frame. After the translocation RUNX1 lost RHD, the fusion product is predicted to be an isoform‐p1 (Ensembl transcript ID: ENST00000300305) with exon1 of RUNX1 influencing the expression of ARID1B. *RUNX1* exon 1 is 5′ noncoding and contributes only to promoter activity and this is fused with *ARID1B* at exon 14. This chimeric structure is predicted to produce an N‐terminally truncated *ARID1B*. Comparative RNA transcription analysis showed a relatively elevated level compared to controls of both *RUNX1* and *ARID1B*. This suggests that *RUNX1::ARID1B* has transcriptional upregulation of *ARID1B* via promoter replacement. Given that the *ARID1B* DNA binding domain spans exons 13–14, truncation starting within exon 14 may alter *ARID1B* SWI/SNF function and result in dysregulation of chromatin accessibility and transcriptional control resulting in accelerated disease. The reciprocal fusion is predicted to be inactive. The truncation of *RUNX1* by the translocation may have resulted in increased transcription of the mutated allele via compensatory mechanism. However, material from diagnostic BM is not available for measuring the RUNX1 transcription to better understand the clinical significance of increased transcription detected in the relapsed specimen. Nevertheless, the absence of wildtype *RUNX1* further enhances differentiation block and disease development.

NGS analysis of diagnostic and relapsed specimens revealed considerable differences in the mutations affecting different genes, but the founding mutation in *IDH1* was retained. Relapsed leukemia had mutations in *ASXL1* and *DNMT3A*. Although they persist through disease progression, alterations in *ASXL1* and *DNMT3A* are believed to be founding alterations, whereas alterations in *RAS* genes and *FLT3*‐ITD are associated with disease progression [30]. Therefore, our observation in this patient points to the possibility that a new clone with mutations in *ASXL1* and *DNMT3A* developed from the founding clone, a cancer stem cell, which upon acquiring *RUNX1*::*ARID1B* led to rapid disease progression and relapse.

Another significant difference between the diagnostic and relapsed specimens is complex karyotypic abnormalities, and these correlated with CNVs involving *JAK2*, *WT1*, and *NF1* detected in NGS. Complex karyotypes are associated with advanced neoplastic conditions; however, the contribution of individual abnormalities to disease biology is not discernible. While mutations in *JAK2*, WT1, or NF1 have been reported in AML and poor prognosis, the clinical impact of deletion of these genes has not been reported.

Flow cytometry results indicated an immature myeloid phenotype with aberrant antigen expression, including partial CD56 expression, a pattern frequently observed in *RUNX1*‐mutated or *RUNX1*‐rearranged AML and had an adverse prognosis [31, 32, 33]. The coexisting truncating *RUNX1* mutation (p.Trp279*) resulting in loss of function and maturation arrest, together with a promoter‐swap *RUNX1::ARID1B* suggest a potential “double‐hit” regulatory mechanism. In this model, insufficiency of normal RUNX1 disrupts normal transcriptional activity of hematopoietic differentiation, while aberrant *ARID1B* expression driven by the *RUNX1* promoter gives rise to a second layer of epigenetic dysregulation through SWI/SNF altered function. Altogether, these changes may act as a compounded pathogenic mechanism arising from concurrent *RUNX1* loss of function and promoter‐driven *ARID1B* abnormal expression.

To the best of our knowledge, this is the first report of AML with “double‐hit” *RUNX1* alterations due to a pathogenic variant p.Trp279* at presentation, and a novel *RUNX1::ARID1B* acquired at relapse. Thus, our study further expands the spectrum of *RUNX1* related chromosomal rearrangements and emphasizes the significance of integrating morphology, cytogenetic, flow cytometry, and NGS data to reveal complex genetic alterations and inform prognostic assessment and may benchmark future therapeutic approaches.

## Author Contributions

R.G. and P.K. designed the study. W.C., S.K.G., and O.W. analyzed morphology. F.F. performed flow cytometry. R.G. and P.K. analyzed cytogenetics and FISH. J.X., R.G., K.S., and L.Y. analyzed NGS and expression data.

## Funding

The authors have nothing to report.

## Ethics Statement

Ethical approval was obtained from the University of Texas Southwestern Medical Center Institutional Review Board (IRB number: STU2024‐0504).

## Consent

The required patient consent was waived by the IRB due to retrospective analysis of clinical laboratory data.

## Conflicts of Interest

The authors declare no conflicts of interest.

## Supporting information


**Table S1:** PCR primers sequence.
**Figure S1:** Peripheral blood smear (100X) and 1B. Bone marrow aspirate (100X): Myeloblasts are large‐sized cells with irregular nuclei, dispersed chromatin, 1–3 prominent nucleoli and scant basophilic cytoplasm. No distinct granules or Auer rods are noted.
**Figure S2:** Flow cytometry Immunophenotype (IP) revealed an acute myeloid leukemia (AML) with a large population of myeloblasts (painted in red) that express CD34, CD33 (partial), and CD56 (not shown) but largely lack CD13 or CD16.
**Figure S3:** Visualization of the c.837G>A, p.W279* and *IDH1* c.394C>G p.Arg132Gly change detected by NGS, as illustrated in the Integrative Genomics Viewer (IGV).

## Data Availability

The data that support the findings of this study are available on request from the corresponding author. The data are not publicly available due to privacy or ethical restrictions.
