# Supplementary material for: Cytogenetic and Molecular Analysis of a “Double‐Hit” RUNX1 Including a RUNX1 p.Trp279* and a Cryptic Novel t(6;21)(q25;q22)/RUNX1::ARID1B in Acute Myeloid Leukemia
Source: Genes Chromosomes Cancer. 2026 Jun 12;65(6):e70148. doi: 10.1002/gcc.70148 (PMC13262018; doi:10.1002/gcc.70148)
Supplement: Supplementary file 1 — Table S1: PCR primers sequence. Figure S1: Peripheral blood smear (100X) and 1B. Bone marrow aspirate (100X): Myeloblasts are large‐sized cells with irregular nuclei, dispersed chromatin, 1–3 prominent nucleoli and scant basophilic cytoplasm. No distinct granules or Auer rods are noted. Figure S2: Flow cytometry Immunophenotype (IP) revealed an acute myeloid leukemia (AML) with a large population of myeloblasts (painted in red) that express CD34, CD33 (partial), and CD56 (not shown) but largely lack CD13 or CD16. Figure S3: Visualization of the c.837G>A, p.W279* and IDH1 c.394C>G p.Arg132Gly change detected by NGS, as illustrated in the Integrative Genomics Viewer (IGV). [file GCC-65-e70148-s001.docx]

**Supplemental Table 1**

PCR primers sequence.

_______________________________________________________________________

PCR Forward TGTAAAACGACGGCCAGTCGATGGCTTCAGACAGCATA

PCR Reverse CAGGAAACAGCTATGACCCATGGAATTGCTGCCAGTTG

_______________________________________________________________________


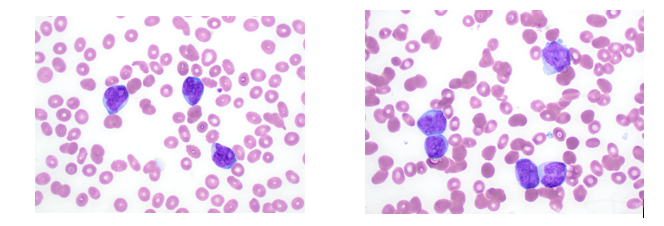


Supplemental Figure 1A. Peripheral blood smear (100X) and 1B. Bone marrow aspirate (100X): Myeloblasts are large-sized cells with irregular nuclei, dispersed chromatin, 1-3 prominent nucleoli and scant basophilic cytoplasm. No distinct granules or Auer rods are noted.


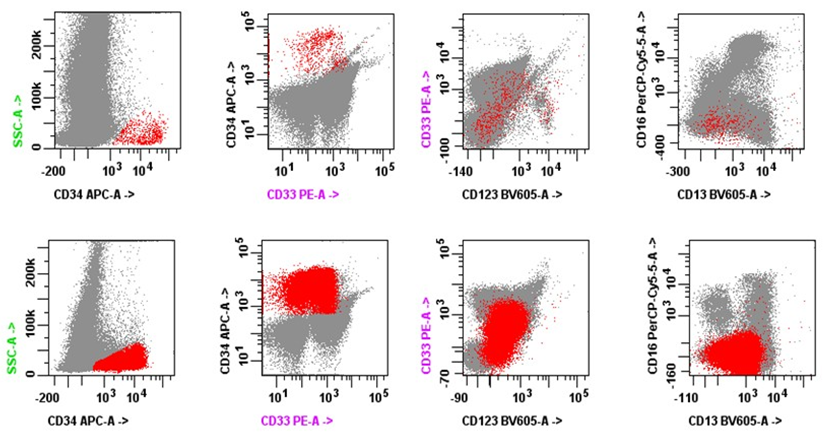


Supplemental Figure 2. Flow cytometry Immunophenotype (IP) revealed an acute myeloid leukemia (AML) with a large population of myeloblasts (painted in red) that express CD34, CD33 (partial), and CD56 (not shown) but largely lack CD13 or CD16


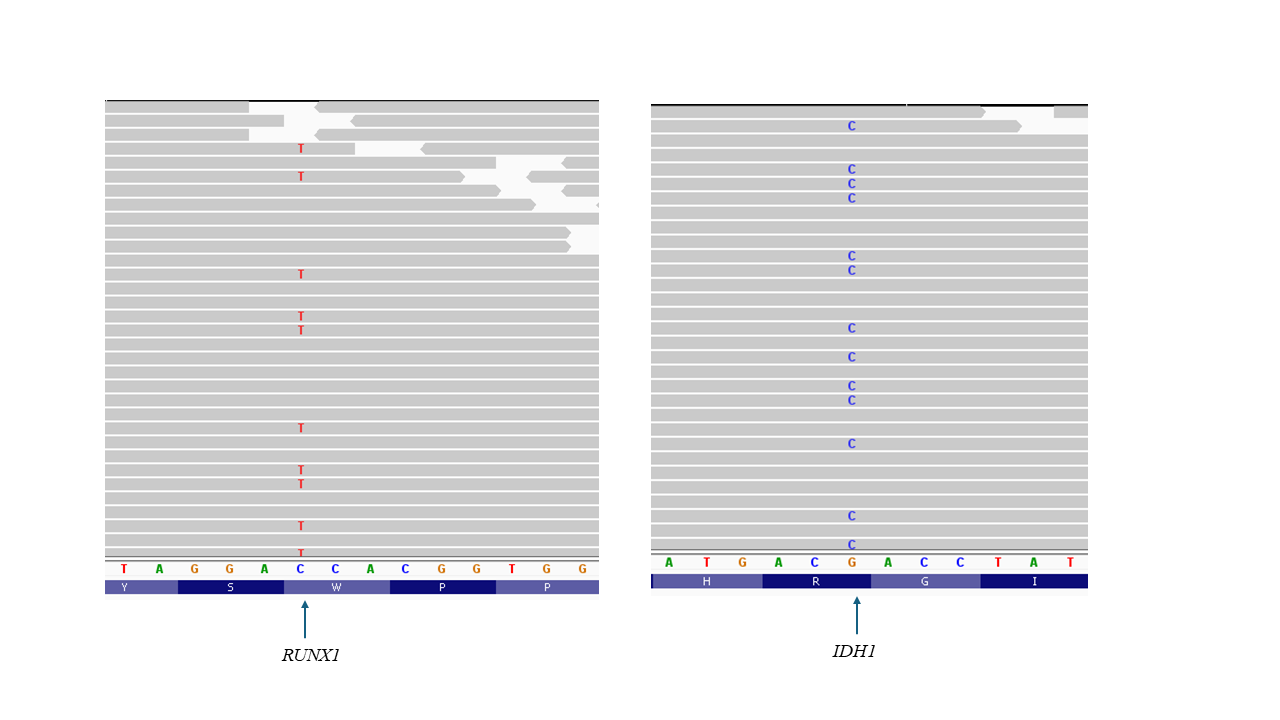


Supplemental Figure 3. Visualization of the c.837G>A, p.W279* and *IDH1* c.394C>G p.Arg132Gly change detected by NGS, as illustrated in the Integrative Genomics Viewer (IGV).
